# Use of days alive without life support and similar count outcomes in randomised clinical trials – an overview and comparison of methodological choices and analysis methods

**DOI:** 10.1186/s12874-023-01963-z

**Published:** 2023-06-14

**Authors:** Anders Granholm, Benjamin Skov Kaas-Hansen, Theis Lange, Marie Warrer Munch, Michael O. Harhay, Fernando G. Zampieri, Anders Perner, Morten Hylander Møller, Aksel Karl Georg Jensen

**Affiliations:** 1 Department of Intensive Care 4131, Copenhagen University Hospital – Rigshospitalet, DK-2100 Copenhagen, Denmark; 2 Section of Biostatistics, Department of Public Health, University of Copenhagen, Copenhagen, Denmark; 3 Clinical Trials Methods and Outcomes Lab, Palliative and Advanced Illness Research Center, Perelman School of Medicine at the University of Pennsylvania, Philadelphia, USA; 4 Department of Biostatistics, Epidemiology, and Informatics, Perelman School of Medicine at the University of Pennsylvania, Philadelphia, USA; 5 HCor Research Institute, São Paulo, Brazil; 6 Department of Critical Care Medicine, Faculty of Medicine and Dentistry, University of Alberta, Alberta, Canada

**Keywords:** Days alive without life support, Days alive out of hospital, Count outcomes, Analysis methods, Statistical models

## Abstract

**Background:**

Days alive without life support (DAWOLS) and similar outcomes that seek to summarise mortality and non-mortality experiences are increasingly used in critical care research. The use of these outcomes is challenged by different definitions and non-normal outcome distributions that complicate statistical analysis decisions.

**Methods:**

We scrutinized the central methodological considerations when using DAWOLS and similar outcomes and provide a description and overview of the pros and cons of various statistical methods for analysis supplemented with a comparison of these methods using data from the COVID STEROID 2 randomised clinical trial. We focused on readily available regression models of increasing complexity (linear, hurdle-negative binomial, zero–one-inflated beta, and cumulative logistic regression models) that allow comparison of multiple treatment arms, adjustment for covariates and interaction terms to assess treatment effect heterogeneity.

**Results:**

In general, the simpler models adequately estimated group means despite not fitting the data well enough to mimic the input data. The more complex models better fitted and thus better replicated the input data, although this came with increased complexity and uncertainty of estimates. While the more complex models can model separate components of the outcome distributions (i.e., the probability of having zero DAWOLS), this complexity means that the specification of interpretable priors in a Bayesian setting is difficult.

Finally, we present multiple examples of how these outcomes may be visualised to aid assessment and interpretation.

**Conclusions:**

This summary of central methodological considerations when using, defining, and analysing DAWOLS and similar outcomes may help researchers choose the definition and analysis method that best fits their planned studies.

**Trial registration:**

COVID STEROID 2 trial, ClinicalTrials.gov: NCT04509973, ctri.nic.in: CTRI/2020/10/028731.

**Supplementary Information:**

The online version contains supplementary material available at 10.1186/s12874-023-01963-z.

## Background

Mortality has traditionally been the primary outcome in most randomised clinical trials (RCTs) in critically ill patients [1]. However, count outcomes such as the number of days alive without life support (DAWOLS; typically including use of mechanical ventilation, vasopressors/inotropes, or renal replacement therapy) and days alive out of hospital (DAOH) are increasingly used [2]. This is motivated by the fact that these outcomes convey more information than binary outcomes such as mortality [3], and their use may reduce the risk of inconclusive RCTs due to the lack of power to reject clinically important effect sizes for mortality [4, 5]. Further, these outcomes not only consider mortality, but also resource use, and as they consider both illness severity (length of periods with life support or in-hospital) and mortality, these outcomes can be considered patient-important [2, 6–8] and may further be associated with other adverse outcomes [6, 7]. Finally, these outcomes easily incorporate occurrent events (e.g., new episodes of life support or readmissions).

However, using, analysing, and reporting DAWOLS, DAOH, and similar outcomes come with challenges compared to those for mortality [2]. These challenges are related to the outcome definitions, including the handling of death and the non-normal distributions, which complicate statistical analyses and may affect the choice of estimand(s) (the quantity estimated in a statistical analysis) and effect measure(s) [2]. Consequently, these outcomes are frequently analysed using various methods, including both regression-based methods and non-parametric tests [2]. Non-parametric tests have previously been recommended [9] and are frequently used [2], but they have important limitations that hamper their usefulness in more complex RCT designs (e.g., multi-arm trials or adaptive platform trials [10]). First, most non-parametric statistical tests primarily provide *P*-values without quantifying effect sizes and uncertainty, which is necessary to assess the clinical importance of a treatment effect. Second, most non-parametric tests either preclude adjustment for covariates (e.g., the Mann–Whitney U/Wilcoxon rank-sum test) or allow only single-variable stratification (e.g., the van Elteren test), and most can only compare two groups at a time [9]. Thus, regression-based methods that allow not only testing null hypotheses, but also estimation of effect sizes may be more appropriate and informative [11] and are increasingly used for these outcomes [2].

In this manuscript, we provide an overview of important conceptual and methodological considerations when using DAWOLS, DAOH, and similar outcomes, including the advantages and disadvantages of different choices, aimed at clinical researchers planning and conducting RCTs using these outcomes. In addition, we discuss different regression-based statistical approaches for analysing these outcomes along with a worked example comparing different models in a real trial dataset.

## Methods

### Scope

We provide an overview of important methodological considerations when using DAWOLS and similar outcomes along with a discussion and comparison of various approaches to statistical analysis. We focus on regression-based statistical methods that allow comparison of more than two treatment arms, adjustment for multiple covariates (e.g., stratification variables, as generally recommended [12], or important prognostic baseline variables, which can increase power [13]), assessment of interactions and heterogeneous treatment effects, quantification of effect measures with measures of uncertainty, and, finally, the use of prior information and ability to generate complete posterior distributions when used in a Bayesian context [10, 14]. We focus on both technical, theoretical, and practical advantages/disadvantages of different choices and models and provide a worked example illustrating and comparing these definitions and models by re-analysing data from the COVID STEROID 2 randomised clinical trial [15].

### COVID STEROID 2 trial data

**Fig. 1:**
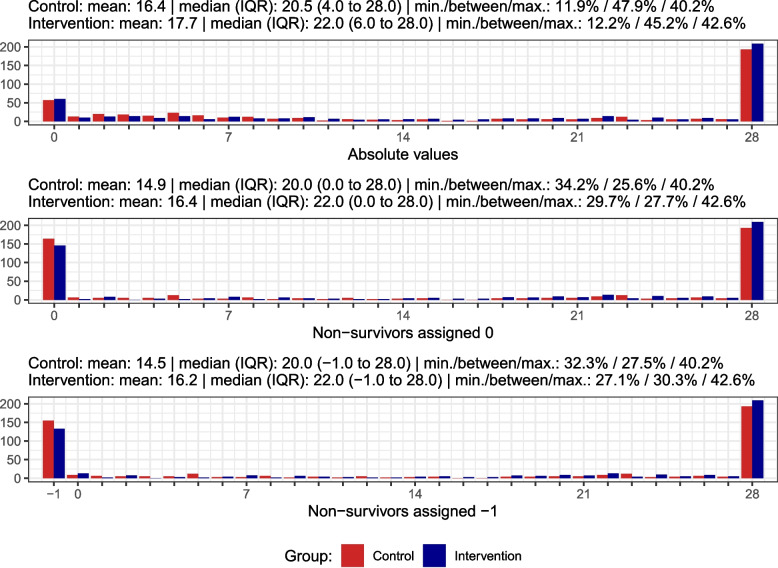
Distributions of days alive without life support (DAWOLS) at day 28 in each treatment group (control group, 6 mg, in red and intervention group, 12 mg, in blue) in the COVID STEROID 2 trial [15]. The distribution of DAWOLS calculated in three different ways are presented: the absolute values (without penalizing death); after assigning the value 0 to non-survivors; and after assigning the value of -1 to non-survivors. Horizontal axes: number of days; vertical axes: number of patients. Abbreviations: IQR: interquartile range, min./between/max.: percentages of patients with the minimum value, values larger than the minimum value and smaller than the maximum value, and the maximum value

We analyse and visualise data from the COVID STEROID 2 trial, which compared a higher (12 mg; intervention) with a lower (6 mg; control) dose of dexamethasone daily for up to 10 days among patients with coronavirus disease 2019 and severe hypoxia defined as the requirement of at least 10 L of oxygen/minute or mechanical ventilation [15]. Randomisation was stratified by site, age less than 70 years and use of invasive mechanical ventilation. DAWOLS was assessed at day 28 with data available for 971 patients. The trial was approved by the Committees on Health Research Ethics in the Capital Region of Denmark (H-20051056), and all additional relevant national/local authorities, conducted in accordance with the Declaration of Helsinki, and all patients or their legal surrogates gave informed consent [15]. Additional details are presented elsewhere [15]. In the primary analyses of the trial, the actual DAWOLS values were used without penalising death, but for these analyses, we assigned a value of 0 (or -1 on the ordinal scale) days to non-survivors. As all data from the COVID STEROID 2 trial were used, no formal sample size calculation was conducted for this study. The outcome data from the COVID STEROID 2 trial using different handling of non-survivors are presented with relevant summary statistics in Fig. 1.

### Outcome operationalisation

**Fig. 2:**
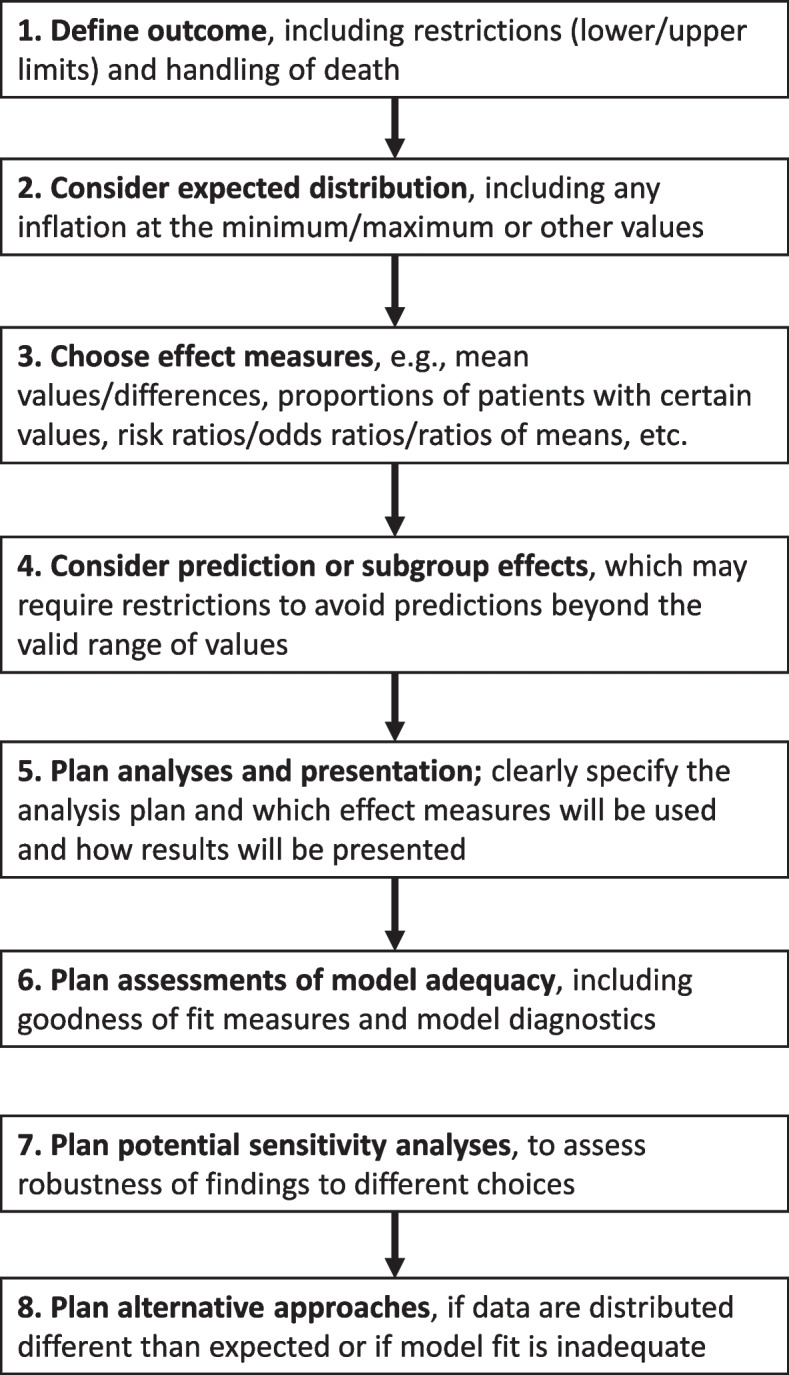
Flowchart illustrating the necessary decisions and considerations when using days alive without life support and similar outcomes in randomised clinical trials. Considerations related to individual steps are further elaborated in the text

Central methodological considerations when operationalising DAWOLS, DAOH, and similar outcomes are summarised in Fig. 2.

First, the outcome definition, including restrictions (e.g., due to maximum follow-up durations) and handling of death, is central. Most commonly, deceased patients are assigned the worst possible value (0 days or a categorical value worse than all possible actual values when analysed as an ordinal outcome) [2], which has previously been recommended to ensure that death is treated as the worst outcome in the analyses [7, 16, 17], even for patients that may be weaned from life support or discharged for shorter periods before they die. In some cases, and especially when longer follow-up periods are used, the actual values (without penalising death) may be preferred [8], and the use of these outcomes without penalising death is also relatively common [2].

Second, the expected outcome distribution should be considered, including whether inflation (peaks) at the minimum and/or maximum values is expected. This may be informed by data from similar, previous studies and will be affected by how death is handled. DAWOLS and DAOH typically have skewed distributions with substantial inflation at the minimum value (as a substantial proportion of the patients either die before getting off life support or discharged, or die within the follow-up period if death is penalised, which usually makes the minimum inflation higher) and sometimes at the maximum value (due to truncation of the follow-up period; consequently, shorter follow-up periods usually increase inflation at the maximum value).

Third, the effect measure (estimand) of primary interest (e.g., mean difference, the difference in proportions of patients with the minimum/maximum/all values, etc.) must be specified. Most commonly, mean differences are used [2], as these weigh values and differences across the full range of the distributions being compared. However, the effect measure of primary interest will depend on the actual trial and trial context.

Fourth, the need for individual predictions or assessment of heterogenous treatment effects (e.g., subgroup analyses) must also be considered, as this may require certain restrictions to prevent predictions beyond the scale of the outcome and may affect the choice of model. For example, if only mean values are of interest, models need not be able to reproduce a distribution of predicted values that resemble the actual outcome distribution.

Finally, an analysis plan should be pre-specified, including which effect measure(s) will be used and how model adequacy will be assessed; whether any potential sensitivity analyses will be conducted; and which alternative approaches may be used if data are distributed differently than expected, if key assumptions are unexpectedly violated, or if the model fit is inadequate. If multiple models are outlined in the analysis plan due to limited knowledge about the distribution of the outcome data, the plan should include a clear strategy for selecting the primary analysis model.

When comparing models and approaches in this study, we focus on DAWOLS defined as the total number of days alive without life support up to a specified maximum number of days, possibly re-scaled to a proportion of the maximum number of days and with dead patients assigned the worst possible value (0 days or a category worse than the lowest possible value when modelled as an ordinal variable, i.e., -1).

### Models

We focus on four specific regression models, with increasing complexity:

1. Linear regression, which primarily models mean values with no lower or upper limits
2. Hurdle-negative binomial regression [18], a two-part count model restricted to non-negative values, consisting of two sub-models:
  - A logistic regression model modelling the probabilities of 0 days
  - A negative-binomial model, an over-dispersed count model modelling the means for all patients with ≥ 1 day with no upper limit
3. Zero–one-inflated beta-regression [19, 20], a three-part model modelling the *proportion* of days, consisting of three sub-models:
  - Two logistic regression models modelling the probabilities of having a proportion of either 0 or 1, and for these patients, the probability of having a proportion of 1
  - A beta regression model (using a beta distribution that may be unimodal, U-shaped, or uniform) modelling the *proportion* of days for all patients with more than 0 and less than the maximum number of days
4. Cumulative logistic regression (also known as proportional odds logistic regression) [21, 22], an ordinal regression model, which models the cumulative probabilities for each category (each possible number of days) restricted to predicting values that appear in the dataset and with the ability to include death as distinct outcome category worse than all other categories

**Fig. 3:**
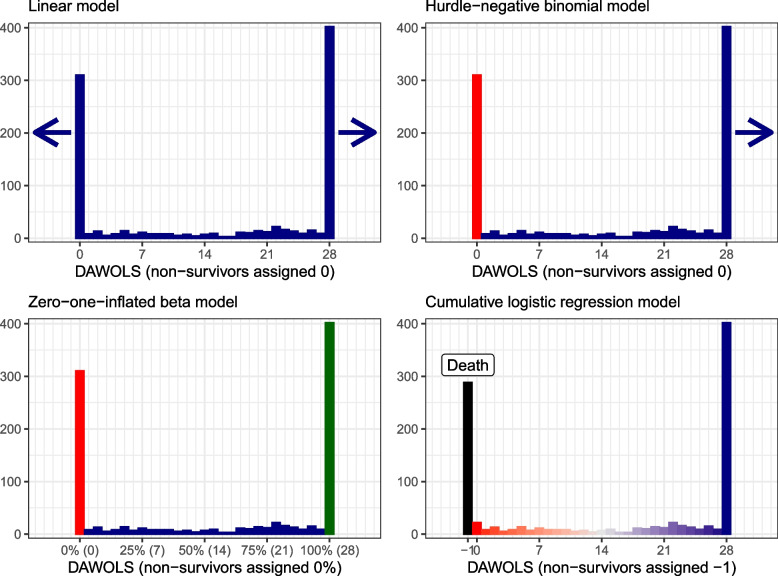
Different models’ handling of days alive without life support (DAWOLS) using COVID STEROID 2 trial data [15]. Horizontal axes: number or proportion of days; vertical axes: number of patients. The linear regression models the mean value of the distribution. No limits are imposed; thus, predictions outside the valid value space in both directions may occur (indicated by the arrows). The hurdle-negative binomial regression models the proportion of patients with exactly 0 days (red) in a logistic regression sub-model and the mean counts for all patients with ≥ 1 day (blue) using a (zero-truncated) negative binomial sub-model. Predictions lower than the valid value space are thus not possible, while predictions above the maximum valid value may occur (indicated by the arrow). The zero–one-inflated beta-model consists of three sub-models and models the *proportion* of DAWOLS. Two logistic regression models estimate the probabilities of having either a proportion of 0 or 1 (0 or 100%, red and green), and the probabilities of a proportion of 1 (100%, green) *conditional* on having either 0 or 1. A beta regression models the proportion of DAWOLS for patients with > 0 and < 1 (> 0% and < 100%, blue) proportion of DAWOLS. The combined model has lower and upper limits corresponding to the valid parameter space; thus, proportions < 0 or > 1 cannot be predicted. The cumulative logistic regression model separately models the probabilities of all distinct values in the dataset as ordinal categories under the proportional odds assumption (Table S1 in Additional file 1). Thus, only values occurring in the dataset will be predicted and specific clinical events (e.g., death) may be included as separate categories, for example, as a category worse than all other values (here -1, black, with all other values visualised using unique colours), although this may complicate prediction on the absolute scale

These models all support as many treatment arms and covariates as desired and can be used to estimate both relative and absolute differences (by calculating either adjusted conditional or marginal estimates for each treatment group), thus enabling interpretation according to clinical importance, which is ideally done on the absolute scale [23]. How the different (sub-)models handle a typical DAWOLS distribution is illustrated in Fig. 3.

Additional details on these models, including their structure, parameters estimated, key assumptions, available effect measures, presentation of results, and benefits and challenges with each model are summarised in Table S1 in Additional file 1.

### Parameter estimation and model comparison

We used Bayesian parameter estimation fitting all models using R software version 4.2.1 (R Core Team, R Foundation for Statistical Computing, Vienna, Austria) and Stan [24] (*cmdstanr* version 2.29.2) through the *brms* [25] R package version 2.18.0. While general considerations would be similar for frequentist methods, Bayesian posterior distributions enable straightforward calculation of all derived quantities with appropriate uncertainty estimates facilitating comparison in these examples. Default (flat or very weakly informative) priors having minimal influence on the results were used for all analyses; all analyses used 4 chains with 10,000 total iterations (2,500 warmup iterations) each, and convergence was assessed using the updated Rhat statistic [26] (≤ 1.01 in all models). Code to fit the models and the exact priors are included in Additional file 1.

The original analyses of the COVID STEROID 2 trial were adjusted for stratification variables [15]; these analyses were unadjusted for simplicity and ease of comparison, as non-collapsibility of some (sub-)models would require marginalisation to facilitate comparability. This would increase unnecessary complexity in this setting [27] but must be considered if the primary analysis model for a future trial is based on a model-comparison based strategy (which should ideally be pre-defined).

To facilitate comparison of different models, we estimated the expected mean number of DAWOLS for each patient/treatment group using predictions for patients in each treatment group calculated by combining predictions from all parts of the hurdle-negative binomial model, the zero–one-inflated beta model (with proportions multiplied by the maximum number of days possible), and the cumulative logistic regression model (with probabilities multiplied by each possible number of days and summed); predicted values were all truncated to the possible outcome space where necessary (including for -1 assigned to non-survivors in the cumulative logistic regression model, which was both replaced with 0 and used as is). We used these values to estimate the mean differences (the most common estimand for these outcomes [2]) and ratios of means to facilitate comparison of all models using the same estimands.

We compared models using *root mean squared errors* (RMSEs) and *median absolute errors* (MAEs) of model predictions compared to the outcome data used to fit the models (on the number of DAWOLS-scale), and, further, by calculating the differences between the predicted and observed mean number of DAWOLS in each treatment group. These summary measures were calculated using the complete posterior distributions and summarised using median posterior values with 95% percentile-based credible intervals (CrIs). As models used different outcome transformations and different distributional families, information criteria-based model comparison was not possible [28].

Model fitness was in particular assessed using graphical posterior predictive checks [29] comparing posterior predictive outcome distributions sampled for each group with the observed outcome distributions. We also used posterior predictive checks to visually compare the distribution of expected (mean) values from each model in each treatment group to the observed group means. In addition, we visually assessed the proportional odds assumption of the cumulative logistic regression model.

Finally, we produced several examples of how the raw data can be presented graphically to provide additional information and to supplement the statistical summary measures by illustrating potential differences across the full range of distributions, inspired by previous RCTs [30–32].

## Results

Measures of model fitness are presented in Table 1.

**Table 1:** Measures of model fitness

**Model**	**Root mean squared errors**	**Median absolute errors**	**Control group difference in predicted vs. observed means**	**Intervention group difference in predicted vs. observed means**	**Control group mean**	**Intervention group mean**	**Mean difference**	**Ratio of means**
Linear	12.6 (12.6 to 12.6)	13.1 (12.0 to 13.9)	0.02 (-1.11 to 1.15)	0.01 (-1.09 to 1.13)	14.9 (13.8 to 16.0)	16.5 (15.4 to 17.6)	1.59 (-0.01 to 3.18)	1.11 (1.00 to 1.23)
Hurdle-negative binomial	12.6 (12.6 to 12.7)	13.1 (12.0 to 13.9)	-0.01 (-1.20 to 1.23)	0.00 (-1.21 to 1.20)	14.9 (13.7 to 16.1)	16.4 (15.2 to 17.6)	1.58 (-0.16 to 3.28)	1.11 (0.99 to 1.24)
Zero–one-inflated beta	12.6 (12.6 to 12.6)	13.2 (12.1 to 13.9)	-0.03 (-1.15 to 1.11)	-0.12 (-1.23 to 0.96)	14.8 (13.7 to 16.0)	16.3 (15.2 to 17.4)	1.49 (-0.09 to 3.05)	1.10 (0.99 to 1.22)
Cumulative logistic (death converted to 0 days in predictions)	12.6 (12.6 to 12.6)	13.0 (12.0 to 13.9)	0.13 (-0.99 to 1.25)	-0.17 (-1.24 to 0.90)	15.0 (13.9 to 16.1)	16.3 (15.2 to 17.3)	1.28 (-0.26 to 2.84)	1.09 (0.98 to 1.20)
Cumulative logistic (death predicted as -1 days)	13.0 (13.0 to 13.0)	13.3 (12.3 to 14.4)	0.14 (-1.01 to 1.30)	-0.17 (-1.27 to 0.93)	14.7 (13.5 to 15.8)	16.0 (14.9 to 17.1)	1.32 (-0.27 to 2.92)	1.09 (0.98 to 1.21)

Model fitness, predictions, and effect estimates for all four models fit to the COVID STEROID 2 trial data [15]; days alive without life support at day 28 was modelled after assigning 0 days to non-survivors in all models except the cumulative logistic model, where non-survivors were assigned -1 (i.e., a category worse than all other categories). Values are presented posterior medians with 95% percentile-based credible intervals. Effect estimates were calculated by predicting the mean value for one patient in each group (as no covariate adjustments were used in these models).

RMSEs were almost identical across models (slightly higher from the cumulative logistic model when predicting -1 for the worst category, corresponding to non-survival); the MAEs were also similar. The differences between predicted and actual mean number of DAWOLS in each group were smallest for the linear, hurdle-negative binomial, and zero–one-inflated beta models and somewhat larger for the cumulative logistic regression model. While the magnitudes of these differences may be of limited importance, estimates in each group were pulled closer in this model. This is likely explained by the proportional odds assumption being somewhat violated (Fig. S1 in Additional file 1).

Predicted means in each group, mean differences, and ratios of means are similarly presented in Table 1. Posterior predictive checks are presented for all model-dataset combinations in Figs. S2-S11 in Additional file 1; increased model complexity generally led to generated data distributions more like the actual data. Predicted mean values from the linear and hurdle-negative binomial models were close to the actual mean values; for the zero–one-inflated beta model, point estimates were slightly lower than the actual values, and for the cumulative logistic model (which had a cumulative odds ratio of 1.21, 95% CrI 0.96 to 1.53), predictions in both groups were closer to each other than in the actual dataset. The linear and hurdle-negative binomial models did not generate data similarly distributed to the COVID STEROID 2 trial dataset, which had substantial inflation at both 0 and 28 days. The zero–one-inflated beta and cumulative logistic regression models generatively replicated the trial data relatively well. Mean values in each group were predicted well by all models, although the cumulative logistic model slightly overpredicted mean values in the control group and underpredicted mean values in the intervention group, drawing the group means closer to each other.

**Fig. 4:**
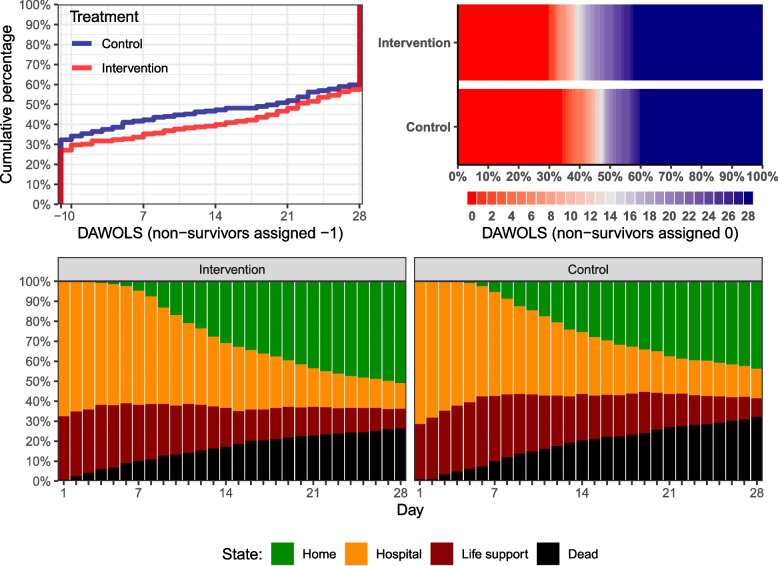
Example visualisations illustrating how visual presentation of days alive without life support or similar outcome data may aid interpretation, using days alive without life support after 28 days from the COVID STEROID 2 trial [15]. Upper left sub-plot: cumulative percentage of patients (vertical axis) with less than or as many days alive without life support as listed on the horizontal axis in each group, after assigning -1 days to non-survivors. This sub-plot shows that patients in the control group had less days alive without life support over the full range of values. Upper right sub-plot:”heat map” visualising the proportion of patients (horizontal axis) with each value in each group after assigning 0 days to non-survivors; this figure is similar to an overturned stacked bar plot, displaying the entire distributions with each unique value having its own colour in a red-to-blue gradient (with colours corresponding to the number of days illustrated in the legend below the plot). Similar to the first sub-plot, this sub-plot shows that, over the full range of values, patients in the control group had fewer days alive without life support. Lower sub-plots: distributions of patients in each possible *state* (vertical axes: alive and home; alive and in hospital; alive, in hospital and on life support; dead) on each day after randomisation (horizontal axes) separately in the two treatment groups. This sub-plot shows additional details compared to the first two: mortality was higher in the control group with more early deaths, and survivors were on life support longer compared to the intervention group

Examples of how the raw data may be visualised are presented in Fig. 4.

## Discussion

We have provided an overview of the necessary methodological considerations and a discussion of four different regression models for assessing skewed and non-trivially distributed outcomes such as DAWOLS and compared these models in real data from the COVID STEROID 2 trial [15]. We have described the models including their key assumptions, structures, data requirements, available effect measures, and aspects related to the presentation of results (Table S1 in Additional file 1). There is no universal best model, and all models have advantages and disadvantages. While the simpler models do not fit the data well enough to mimic the input data, they adequately estimated group means, are simpler to interpret, and are easier to use in a Bayesian context with informative priors. In contrast, the more complex models provided better fits to the data and were better at generating new, similar data, although none were perfect. The more complex models can describe separate components of the outcome (i.e., the probability of having 0 or more DAWOLS), which may be relevant in some settings. However, this comes at increased complexity, increased uncertainty, increased difficulty related to the use of meaningful priors, and in some cases, somewhat larger errors for estimated mean values.

Linear regression is easy to interpret, simple to work with, and may be used if separate estimates for specific counts, such as minimum/maximum values, are not needed. While the assumption of normally distributed residuals (Table S1 in Additional file 1) will usually be violated when modelling outcomes such as DAWOLS, means and uncertainty metrics (confidence/credible intervals) may be adequately estimated for larger samples, due to the central limit theorem [33, 34] in the frequentist setting, if Bayesian posterior distributions are used, or if bootstrapping [35] is used to estimate robust standard errors. Thus, linear regression often works well even with large departures from normality [36] and, in practice, also often for probability and proportion data [37]. As an alternative, quantile regression [38, 39] may be used to estimate (adjusted) medians. While quantile regression does not have the same distributional assumptions as linear regression, it is computationally more complex and requires relatively more data to adequately estimate uncertainty and may thus not work as well in smaller samples [39].

Multi-part models, including hurdle-negative binomial and zero–one-inflated beta models may better generatively replicate data and provide combined and separate estimates for each sub-model. This may be important if a treatment has opposite effects on, e.g., mortality and the duration of organ support in survivors/non-survivors, leading to opposing differences in the probabilities of 0 days and the mean number of days in all other patients. Such opposing effects may hamper the interpretation of the combined estimate and decrease power [17]; however, this may also be assessed by analysing mortality separately as is usually done and recommended [2]. Zero–one-inflated beta regression may be theoretically preferable to hurdle-negative binomial models if some inflation at the maximum value is expected; however, hurdle-negative binomial models are simpler to work with and estimated the group means better in these examples. Consequently, even if some inflation at the maximum value is expected, a hurdle model may be an appropriate choice if the primary interest revolves around modelling the proportion of values of 0 and > 0, and hurdle models may also offer increased precision compared with three-part models due to the use of one less sub-model. Alternatively, longer follow-up durations may be chosen to limit the inflation to the maximum value. Similar models not covered here include hurdle-Poisson models (Poisson models are less flexible than negative binomial models, which may lead to inferior fits [40]), hurdle-log-normal models suitable for modelling non-negative continuous (non-count) data, and zero-inflated negative binomial/Poisson models (similar to hurdle models, but model 0 as coming from two separate processes and thus complicates interpretation). Finally, beta-binomial models (an over-dispersed binomial model) may also be considered for count outcomes with maximum values [40–42]. This model may provide fits like the zero–one-inflated beta regression with higher precision (as it does not consist of multiple sub-models) but without the ability to separately estimate minimum/maximum values. Alternatively, an ordinal beta regression model has recently been proposed and may likewise be considered [43].

The cumulative logistic regression model assessed has the least distributional assumptions and may fit data well overall. However, the ordinal odds ratio is somewhat difficult to interpret, as it is not on an absolute or clinically interpretable scale. Thus, defining a clinically relevant difference is difficult, unless mean values are estimated by combining the probabilities of all counts in the data. The central assumption of this model (i.e., odds being proportional) may not hold, and, from a clinical perspective, treatment effects may plausibly be different, at least in magnitude, on mortality and the number of days alive and without life support in survivors. Thus, while violations of the proportional odds assumption do not necessarily invalidate the model (Table S1 in Additional file 1) [44], they may affect results, especially if means or other absolute effect measures are calculated. In such situations, the use of proportional odds models may lead to under-/overestimated group means (and consequently incorrect mean difference estimates), as seen in these analyses. Of note, the proportional odds assumption may be relaxed using the (constrained) partial proportional odds model [45], although this further increases the complexity of both modelling and interpretation.

In addition to the overall statistical model used, detailed graphical presentation of the overall and daily raw data and presentation of probabilities of minimum/maximum values and overall mortality for the same period of follow-up is advisable to ease interpretation and to ensure that opposing directions of effects on mortality and the number of days in survivors are not present, as the combined estimate may not be clinically meaningful if this is the case [17]. Different visualization options are available; methods that visualize the distributions of DAWOLS (e.g., the two upper sub-plots in Fig. 4) are relatively easy to interpret and quickly provides an overview and an impression about whether different directional effects are present. More details may be conveyed using more complex plots (e.g., the lower sub-plot in Fig. 4 illustrating the proportions of patients in different states each day), however, the added complexity require more scrutiny to fully interpret.

### Strengths and limitations

This study comes with several strengths. First, we have provided an overview (Fig. 2) of the central methodological considerations and decisions, including each model’s technical, theoretical, practical, and interpretational aspects. This is important in practice, as interpretability may be a valid reason to choose one model over another, especially if individual predictions are not needed. Second, we used Bayesian methods and graphical posterior predictive checks to provide an intuitive and visual interpretation of how well models fit the complex distributions and allow easy calculation of derived, combined effect estimates (including uncertainty) for multi-part models. While these analyses were conducted in a Bayesian context, most considerations discussed in this manuscript apply equally to the frequentist setting.

The study comes with limitations, too. First, we only compared models in a single dataset; we have thus provided an example, but relative model performance and goodness-of-fit in other datasets may differ somewhat. Comparing model performance in multiple, real datasets would be valuable moving forward and is likely preferable to simulation-based model comparison, as the complexity of the distributions of DAWOLS and DAOH is a challenge with results likely to depend substantially on the data-generating model used in such simulations. Second, we considered a limited number of models and only focus on regression models, without considering non-parametric tests due to the limitations outlined in the background. Time-to-event/survival models have been used in some RCTs employing variants of DAWOLS (e.g., RCTs focusing on mechanical ventilation where all patients are ventilated at baseline [46]), but these methods have not been covered here, as they are often not suitable, e.g., when not all patients start on life support or when a substantial number of patients get on/off life support at multiple times during admission, or where hospital readmission after initial discharge is common or relevant. More complex models such as Gaussian processes and kernel density estimators, which may provide better fits to data, and longitudinal Markov/state-transition models could be considered, all of which use all daily available information without collapsing the results into a single number of days. Importantly, the considered models are all well-documented, relatively easy to use and interpret, and are implemented in stable and well-documented software packages [25]. Finally, we have not in detail discussed the general benefits and disadvantages of DAWOLS or similar types of outcomes and their different definitions; this has been done elsewhere [2, 8, 17, 46] and is beyond the scope of the present manuscript.

### Recommendations

As there are potential advantages and disadvantages with all models used to analyse DAWOLS and similar count outcomes, it is paramount to pre-specify a statistical analysis plan based on careful considerations of the expected data distributions, clinically relevant effect measures of interest, and the important benefits and trade-offs with the model chosen. As illustrated previously, choosing different distributions may affect results and their interpretation [40]. Thus, the approach must be pre-specified, and ideally include a primary plan and a plan for assessing model assumptions and fitness with alternative approach(es) to be invoked if the primary proves inappropriate [34, 40]. Further, sensitivity analyses using different approaches may be considered to assess the influence of model choice. Finally, we strongly recommend that detailed outcome data are presented and considered (graphically and/or as separate components) to ease interpretation and to avoid misleading conclusions if opposing effects on different components cancel out in whole or in part [17]. Presentation of separate mortality data at the same follow-up time point is especially important [2].

## Conclusions

In conclusion, we have discussed important methodological considerations when analysing DAWOLS and similar count outcomes. We have discussed and assessed several regression models that are extendable and suitable for both simple and complex RCTs, enable estimation of treatment effects regardless of the number of treatment arms, and allow for adjustment for several covariates. The discussed models all come with advantages and disadvantages, and while no model is globally recommended, this manuscript should help clinical trialists, statisticians, and other researchers make an informed choice.

## Supplementary Information

**Additional file 1. **A table summarising the models considered and additional plots, including assessment of the proportional odds assumption for the cumulative logistic model and posterior predictive checks for all models, can be found in Additional file 1.pdf.

## Data Availability

Fully de-identified individual participant data from the COVID STEROID 2 trial will be shared after approval by the trial Management Committee after reasonable request to the trial Management Committee. Analysis code for this project may be obtained by request to the corresponding author.
