# Supplementary material for: Use of days alive without life support and similar count outcomes in randomised clinical trials – an overview and comparison of methodological choices and analysis methods
Source: BMC Med Res Methodol. 2023 Jun 14;23:139. doi: 10.1186/s12874-023-01963-z (PMC10266319; doi:10.1186/s12874-023-01963-z)
Supplement: Supplementary file 1 — Additional file 1. A table summarising the models considered and additional plots, including assessment of the proportional odds assumption for the cumulative logistic model and posterior predictive checks for all models, can be found in Additional file 1.pdf. [file 12874_2023_1963_MOESM1_ESM.pdf]

aUse of days alive without life support and similar count outcomes in randomised clinical trials – an overview and comparison of methodological choices and analysis methods

Anders Granholm[1], Benjamin Skov Kaas-Hansen[1,2], Theis Lange[2],
Marie Warrer Munch[1], Michael O. Harhay[3,4], Fernando G. Zampieri[5,6],
Anders Perner[1], Morten Hylander Møller[1], Aksel Karl Georg Jensen[2]

Additional file 1

Supplementary tables and figures

[1] Department of Intensive Care 4131, Copenhagen University Hospital – Rigshospitalet, Copenhagen, Denmark

[2] Section of Biostatistics, Department of Public Health, University of Copenhagen, Copenhagen, Denmark

[3] Clinical Trials Methods and Outcomes Lab, Palliative and Advanced Illness Research Center, Perelman School of Medicine at the University of Pennsylvania, Philadelphia, USA

[4] Department of Biostatistics, Epidemiology, and Informatics, Perelman School of Medicine at the University of Pennsylvania, Philadelphia, USA

[5] HCor Research Institute, São Paulo, Brazil

[6] Department of Critical Care Medicine, Faculty of Medicine and Dentistry, University of Alberta, Alberta, Canada

Table of contents

Table S1. Summary of models considered 2

Model syntax and priors 8

Figure S1. Assessment of the proportional odds assumption in cumulative logistic model 12

Posterior predictive check plots (Figures S2-S11) 14

References 19

Table S1. Summary of models considered

Model	Data format, model structure/parameters/important assumptions	Effect measures and presentation	Advantages and disadvantages
Linear regression	Raw count data – not truncated at the minimum and maximum values.<br>Models mean and standard deviation of residuals – mean difference modelled directly. Assumes normally distributed residuals [1].	Conditional/marginal mean values** in each group.<br>Mean difference.<br>Measures derived from the above values, e.g., ratio of means.	Unlikely to adequately fit the typical distribution of days alive without life support (and similar outcomes) and thus unable to generate new, similar data from the model.<br>Works for outcomes with zero-inflation and values below zero (e.g., some HRQoL tools [2]), but interpretation of results is hampered if death is considered a worse outcome than 0 days [3].<br>May adequately estimate the mean difference with uncertainty for larger sample sizes (according to the central limit theorem [1]) and can adequately assess uncertainty even if the residuals are not normally distributed if Bayesian posterior distributions for the means/mean differences are analysed, or if bootstrapping or robust standard errors are used in the frequentist setting.<br>May provide implausible predictions in some cases due to the lack of upper and lower truncation.<br>Quick to estimate and easy to interpret parameters; only one set of covariate estimates needed.<br>Easy to set interpretable priors on all parameters if used in a Bayesian context.<br>Does not separately model minimum/maximum values, so if these are of specific interest, other methods or separate analyses are needed.

**Model**	**Data format, model structure/parameters/important assumptions**	**Effect measures and presentation**	**Advantages and disadvantages**
Hurdle-negative binomial regression [4]	Raw count data (whole numbers without decimals) – only allows non-negative values, but no truncation at any upper maximum value.<br>Two-part* model:<br>1) Logistic regression model modelling the probability of 0 days alive without life support.<br>2) Zero-truncated negative-binomial model: over-dispersed count model, modelling the mean number of days alive without life support for all patients with values larger than zero.	Conditional/marginal probabilities of 0 days in each group**; odds ratio for having 0 days; derived measures including risk ratios or risk differences for having 0 days.<br><br>Conditional/marginal mean values** in each group; mean difference; rate ratio for those with >0 days.<br>Overall conditional/marginal mean values** in each group; mean difference/rate ratio or ratio of means from the combined parts of the model.	Lower truncation at 0; no upper truncation. Can adequately model excess zeroes, but may not be able to adequately fit or generate similar data with substantial inflation at the maximum value.<br>Does not work for outcomes with both zero-inflation and values below zero (e.g., some HRQoL tools [2] or if death is considered a worse outcome than 0 days without life support [3]).<br>May adequately estimate the combined mean difference with uncertainty for larger sample sizes and can adequately assess uncertainty if Bayesian posterior distributions for the means/mean differences are analysed, or if bootstrapping or robust standard errors are used in the frequentist setting.<br>May provide implausible predictions in some cases due to the lack of upper truncation.<br>Two components, more complex and time-consuming to estimate than a linear model; two sets of estimates per covariate needed, which can increase uncertainty, especially in smaller samples.<br>Relatively straightforward to set interpretable priors on all parameters if used in a Bayesian context, but more difficult than a linear regression and incorporating previous evidence may be difficult if not reported as separate components for the two parts in previous research.

**Model**	**Data format, model structure/parameters/important assumptions**	**Effect measures and presentation**	**Advantages and disadvantages**
Zero-one-inflated beta regression [5, 6]	Count data scaled as proportions relative to the maximum value [0-1] – truncated at the minimum/maximum values, results can be back-transformed to counts by multiplication with the maximum value.<br>Three-part* model:<br>1) Logistic regression modelling the probability of zeroes/ones.<br>2) Logistic regression modelling the probability of 1 conditional on the value being 0 or 1.<br>3) Beta regression modelling all values between 0 and 1; the beta distribution can take a number of shapes (including uni- and bimodal shapes, the latter U-shaped).	Conditional/marginal probabilities of 0 and 1** (or 0 and 1, plus 1 conditional on being 0 or 1).<br>Conditional/marginal mean proportions in each group**, can be rescaled to the actual scale (number of days).<br>Overall mean difference/rate ratio or ratio of means, from the combined parts of the model.	Values restricted to valid proportions; both upper and lower truncation.<br>Can adequately model excess minimum and maximum values; all predicted values will be in the valid range.<br>Does not work for outcomes with zero-inflation and values below 0 (e.g., some HRQoL tools [2] or if death is considered a worse outcome than 0 days without life support [3]).<br>Flexible distribution but may not always fit the data perfectly.<br>Three components, more complex and time-consuming to estimate than simpler models; three sets of estimates per covariate are needed, which can increase uncertainty, especially in smaller samples.<br>Possible to set interpretable priors on all parameters if used in a Bayesian context, but more difficult than for simpler models and incorporating previous evidence may be difficult if not reported as separate components for the three parts.

Model	Data format, model structure/parameters/important assumptions	Effect measures and presentation	Advantages and disadvantages
Zero-one-inflated beta regression (continued)	Technically, the zero-one-inflated beta model is a two-hurdle model, as 0 and 1 values are only estimated by the logistic regression sub-models.<br>If there are no maximum values (no values of 1) in the data, the model can be reduced to a zero-inflated beta model.		

Model	Data format, model structure/parameters/important assumptions	Effect measures and presentation	Advantages and disadvantages
Cumulative logistic regression (ordinal regression/proportional odds logistic model) [7, 8]	Each unique value in the dataset considered a distinct ordinal category. May include death as a category worse than 0 [3]; this may provide interpretational benefits, especially for shorter follow-up times, where most would likely consider death as a worse outcome than being on organ support for the entire period and surviving.<br>Semiparametric model – no distributional assumptions, but assumes *proportional odds*: that the effect of the treatment and covariates is the same across all cut-points (moving from each level of the ordinal scale to the next).<br>Can be considered a generalisation of common non-parametric tests that allows adjustment [7, 9].	Odds ratio for the treatment difference.<br>Can also calculate the conditional/marginal probabilities** of each value in the dataset for all or reference patients**; these can be used to calculate cumulative probabilities of, i.e., having a value ≤*X* or by summarising the probabilities of all individual values with the actual values to calculate marginal/conditional** means, mean differences and rates.	No distributional assumptions – can also model values below 0 (e.g., if HRQoL is assessed [2] or if death is included as a distinct outcome worse than 0 days [3]).<br>Primary result is an odds ratio – easier-to-interpret results may be derived from the model (although it may be necessary to, e.g., convert distinct event categories, such as death modelled as -1, to the valid range of values, e.g., 0).<br>If the proportional odds assumption is violated, the result can still be seen as a valid average of the odds ratios at the different cut-points [7, 9], but may then not generate data that closely mimics the input data and calculation of derived estimates (e.g., on the absolute scale) may be incorrect.<br>Many terms to estimate as separate intercepts have to be estimated for each value of the outcome variable (minus one) so more time-consuming to estimate than simpler models.<br>Difficult to set easily interpretable priors on all parameters if used in a Bayesian context, and incorporating previous evidence may be difficult or not possible unless reported as separate components.

Description of central features of the four models assessed; of note, all models support adjustment and multiple groups as needed. Only important assumptions are listed; all models discussed assume independence of observations and linearity on the appropriate scale.

* For multi-part models, different covariates and model formulas can be used for the individual components; this is not recommended for modelling data from randomised controlled trials, as different interventions may affect all components. In multi-part models, combined probabilities for the different effect estimates from each part of the model can be calculated, which may aid in interpretation.

** Estimates in each group may be derived using marginalisation over the entire sample or as conditional effect estimates using a reference patient approach, i.e., by estimating expected values for patients in each group with categorical covariates set to their most common values and continuous variables to their mean/median values.

Abbreviations: HRQoL: health-related quality of life.

Model syntax, and priors

In this section, the syntax required to fit the models and the used link functions using the *brms* R package, and the complete priors used (all flat or very vaguely informative, corresponding to the current default priors in *brms*). In the syntax listed … denotes non-general parts of the model calls, *dawols_dead0* denotes days alive without life support to day 28 with the value 0 assigned to non-survivors, *dawols_dead0_prop* corresponds to *dawols_dead0* but scaled to a proportion (by division with the maximum possible value, 28), *dawols_deadm1_fct* denotes the days alive without life support to day 28 with -1 assigned to non-survivors and encoded as a categorical variable. As the *center* argument was not specified (see *brmsformula* documentation), distributional parameters were centred and priors are thus on the centred parameters.

Linear regression

Syntax: *brm(brmsformula(dawols_dead0 ~ treatment), family = gaussian(link = "identity"), …)*.

Priors:

- *treatment*: a flat, i.e., improper/uniform prior on the treatment effect.

- *intercept:* a *student_t(3, 22, 8.9)* prior, i.e., a Student's T-distribution prior with 3 degrees of freedom, mean of 22 and standard deviation of 8.9.

- *sigma*: a *student_t(3, 0, 8.9)* prior for the sigma parameter with a lower boundary of 0, i.e., a half-Student's T distribution prior.

Hurdle-negative binomial regression

Syntax: *brm(brmsformula(dawols_dead0 ~ treatment, hu ~ treatment), family = negbinomial(mu = "log", shape = "identity", hu = "logit"), …)* with the two parts of the formula specifying that both parts of the model (with *hu* denoting the logistic regression sub-model) are assumed to vary with the treatment allocation (of note, the default link function for the *shape* auxiliary parameter in *brms* is an identity link if not modelled separately, but a log link if a specific model formula is provided for this parameter).

Priors:

- *treatment*: flat, i.e., improper/uniform prior on the treatment effect in both sub-models.

- *intercept:* a *student_t(3, 3.1, 2.5)* prior on the intercept part of the zero-truncated negative-binomial sub-model.

- *intercept, hu:* a *logistic(0, 1)* prior on the intercept part of the logistic regression sub-model, i.e., a logistic prior with mean of 0 and standard deviation of 1.

- *shape*: a *gamma(0.01, 0.01)* prior for the shape (phi) parameter of the zero-truncated negative-binomial sub-model, i.e., a gamma prior with alpha and beta parameters of 0.01 and a lower boundary of 0.

Zero-one-inflated beta regression

Syntax: *brm(brmsformula(dawols_dead0_prop ~ treatment, zoi ~ treatment, coi ~ treatment), family = zero_one_inflated_beta(mu = "logit", phi = "identity", zoi = "logit", coi = "logit"), …)* with the three parts of the formula specifying that all three parts of the model (with *zoi* denoting the sub-model modelling the probability of having proportions of 0 or 1 and *coi* denoting the sub-model modelling the probability of having proportions of 1 conditional of having a proportions of either 0 or 1) are assumed to vary with the treatment allocation (of note, the default link function for the *phi* auxiliary parameter in *brms* is an identity link if not modelled separately, but a log link if a specific model formula is provided for this parameter).

Priors:

- *treatment*: flat, i.e., improper/uniform prior on the treatment effect in all three sub-models.

- *intercept:* a *student_t(3, 0, 2.5)* prior on the intercept part of the beta regression sub-model.

- *intercept, zoi* and *coi: logistic(0, 1)* prior on the intercept part of the two logistic regression sub-models, i.e., a logistic prior with mean of 0 and standard deviation of 1.

- *phi*: a *gamma(0.01, 0.01)* prior for the phi parameter of the beta regression sub-model, i.e., a gamma prior with alpha and beta parameters of 0.01 and a lower boundary of 0.

Cumulative logistic regression (ordinal regression/proportional odds logistic model)

Syntax: *brm(brmsformula(dawols_deadm1_fct ~ treatment), family = cumulative(mu = "logit", disc = "identity", threshold = "flexible"), …).*

Priors:

- *treatment,* all specific *intercepts*: flat, i.e., improper/uniform prior on the treatment effect and all intercepts in the model.

Figure S1. Assessment of the proportional odds assumption of the cumulative logistic model

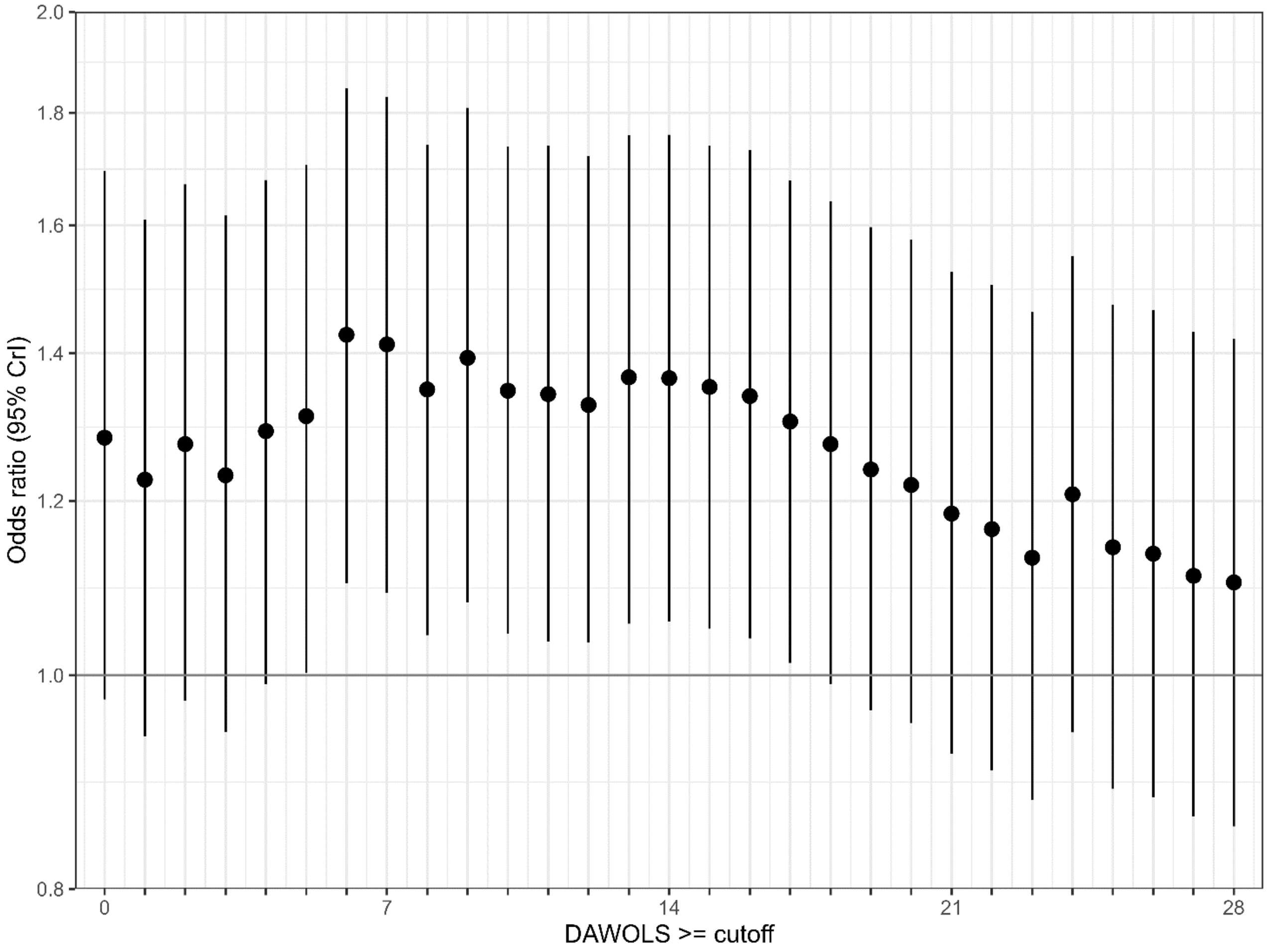

This figure was used to visually assess the proportional odds assumption in the cumulative logistic (proportional odds logistic) regression model. Separate logistic regression models were fitted for each potential dichotomization of the outcome (days alive without life support at day 28, with non-survivors assigned the value -1), i.e., for having ≥1 days, ≥2 days, etc. The odds ratios with 95% credible intervals (CrIs) for each cutoff are displayed; odds ratios >1 indicate that the intervention group had higher probabilities of more days alive without life support. If the proportional odds assumption holds, the odds ratios should be similar at all cut-offs; different odds ratios at different cut-offs implies violations of the proportional odds assumption. As completely identical odds ratio estimates and CrIs at all intervals are unlikely, potential differences should be interpreted in light of the uncertainty and overall tendencies and large differences (especially if not only different in magnitude but also in direction) are more indicative of important violations than slight variations.

Posterior predictive check plots

The following plots illustrate the posterior predictive checks [10] for all models. The plots labelled 'densities' present posterior predictions for all patients in the COVID STEROID 2 trial [11] dataset for the two treatment groups; of note, these overlain density plots give a good overall view of the distributions, but the smoothing may lead to some visual artefacts primarily close to the minimum/maximum values. The plots labelled 'means' present the posterior expected means for all patients in the COVID STEROID 2 trial [11] dataset for the two treatment groups. The dark blue lines/bars represent the actual data, whereas the light blue lines and bars represent the posterior predictions/expected means.

Figure S2. Posterior predictions from the linear model, densities in each group

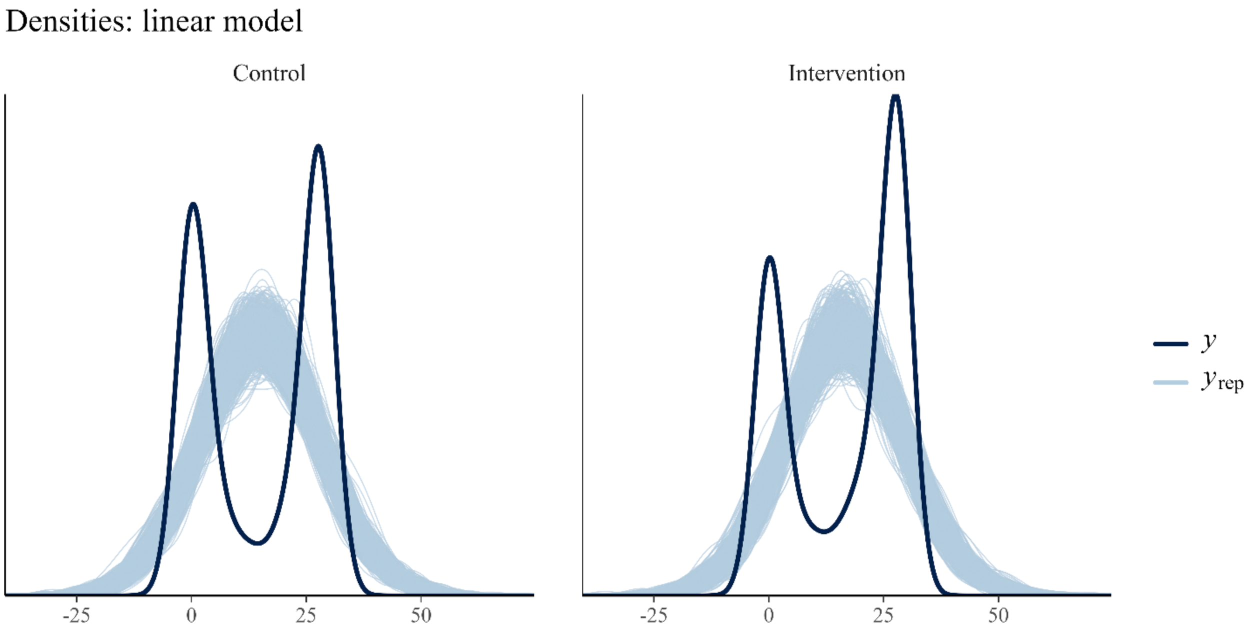

Figure S3. Posterior expected means from the linear model, means in each group

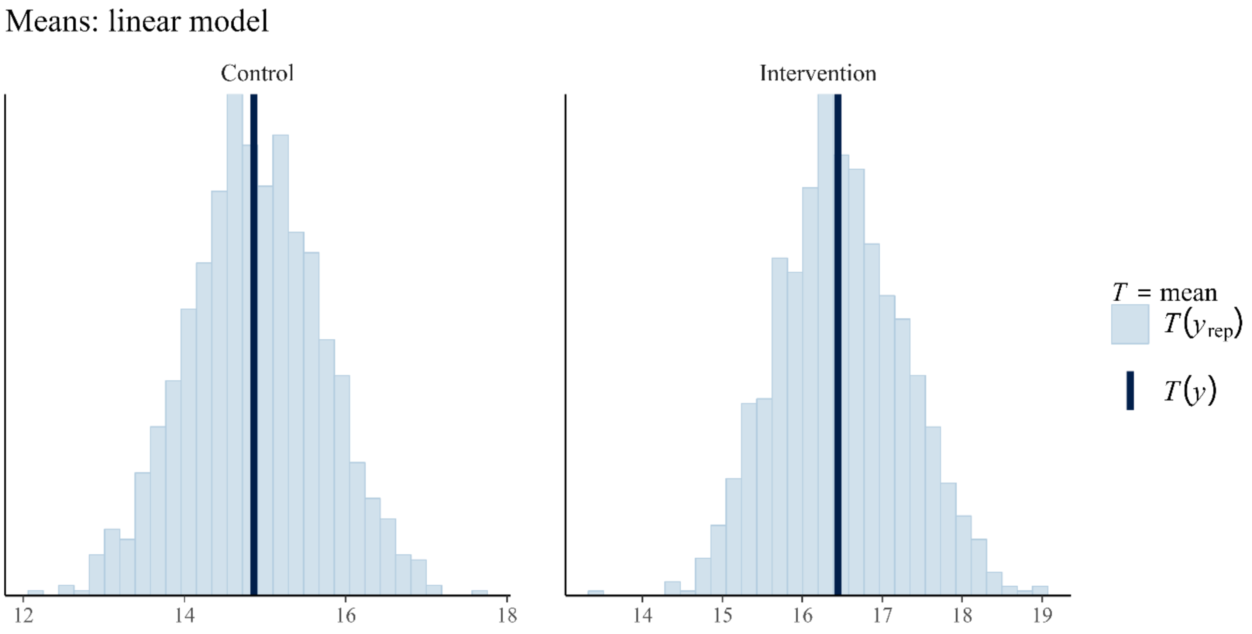

Figure S4. Posterior predictions from the hurdle-negative binomial model, densities in each group

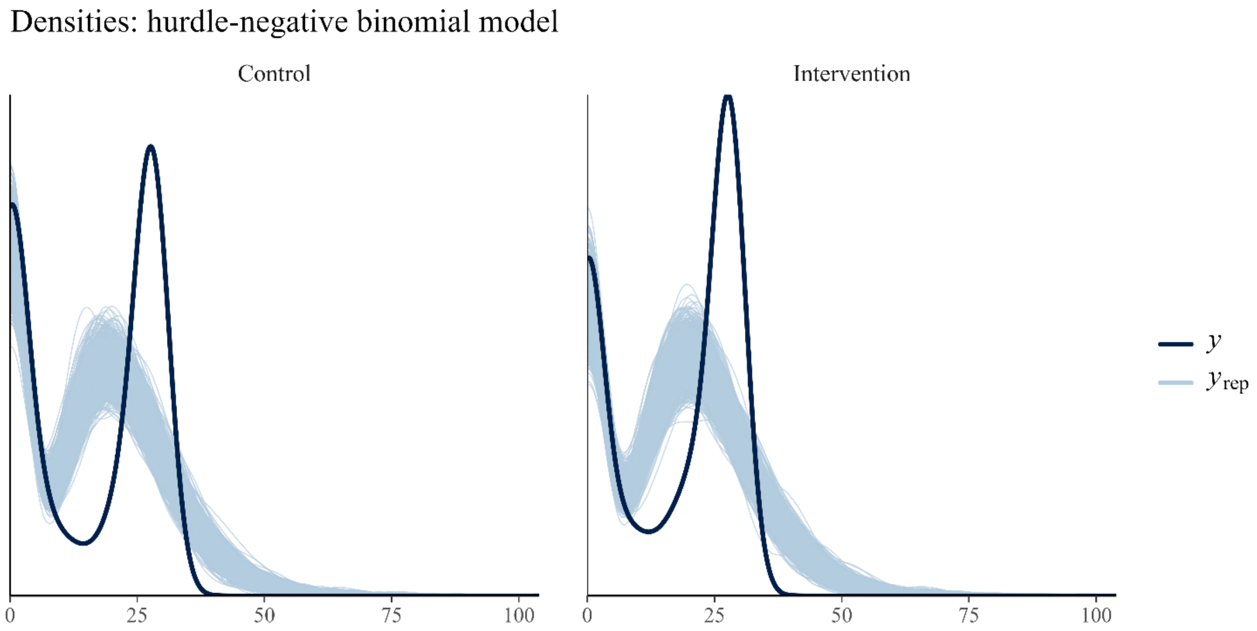

Figure S5. Posterior expected means from the hurdle-negative binomial model, means in each group

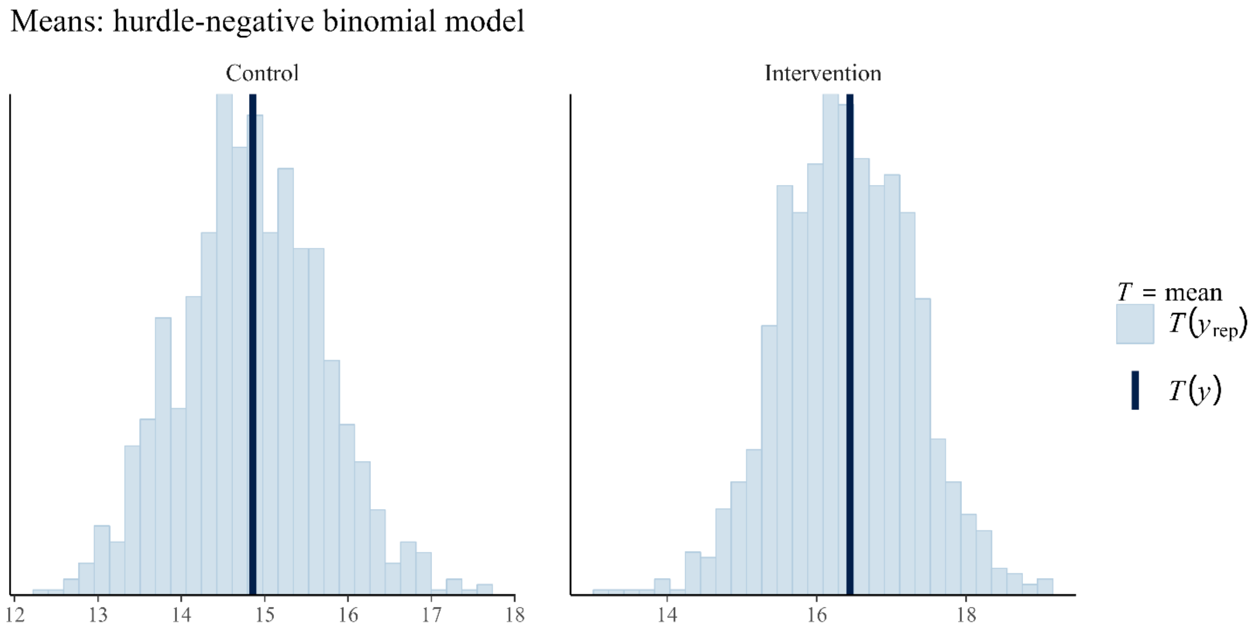

Figure S6. Posterior predictions from the zero-one-inflated beta model, densities in each group

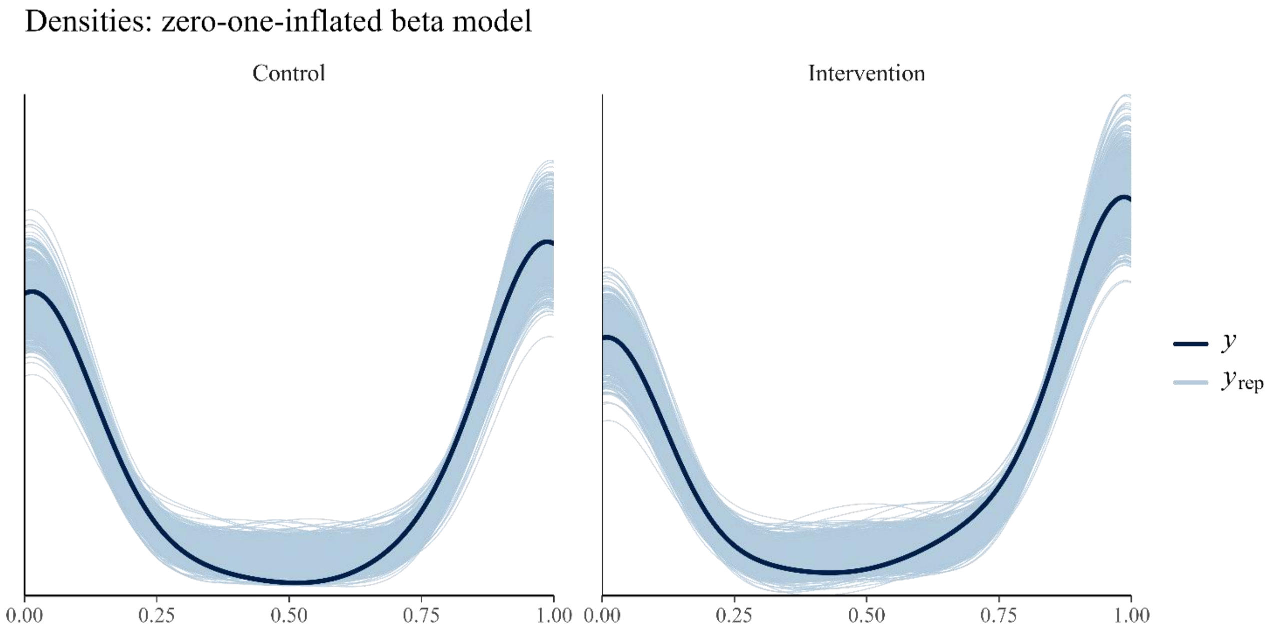

Figure S7. Posterior expected means from the zero-one-inflated beta model, means in each group

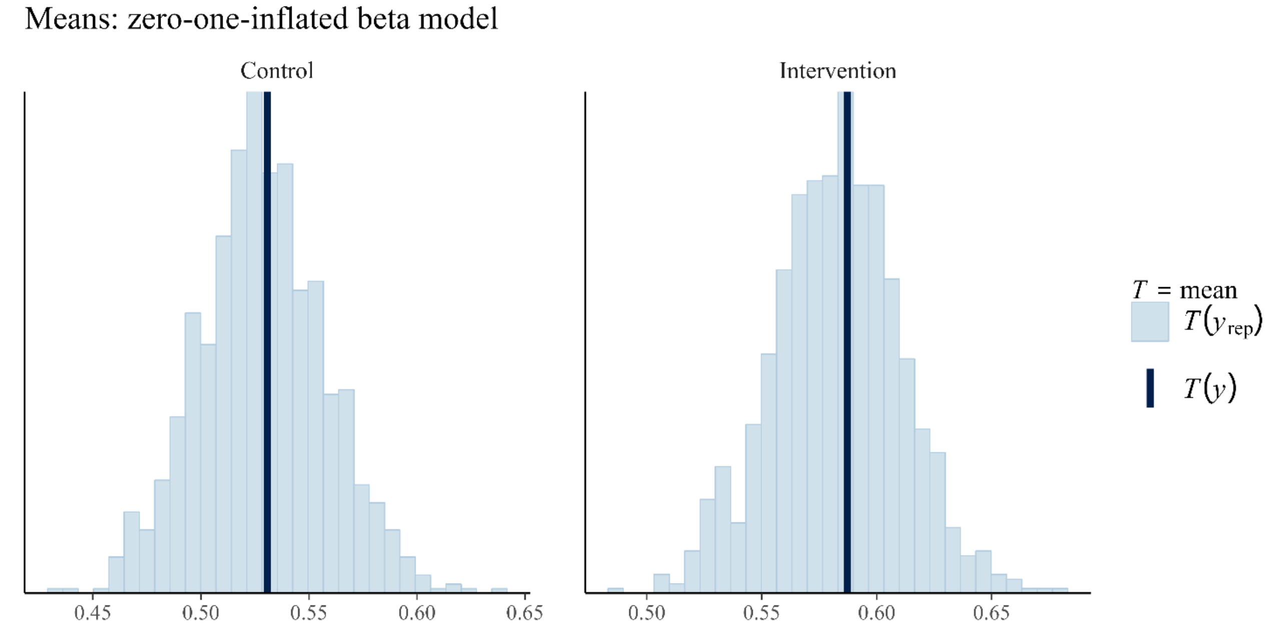

Figure S8. Posterior predictions from the ordinal (cumulative/proportional odds logistic regression) model, densities in each group, 0 predicted instead of -1 for worst category

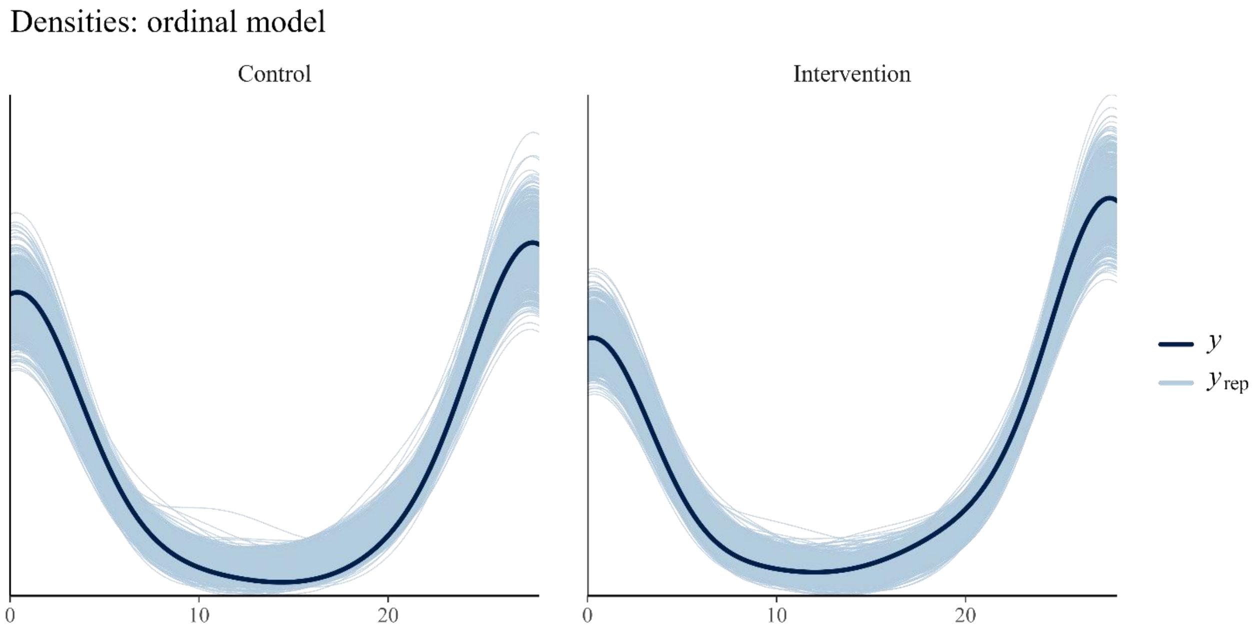

Figure S9. Posterior expected means from the ordinal (cumulative/proportional odds logistic regression) model, means in each group, 0 predicted instead of -1 for worst category

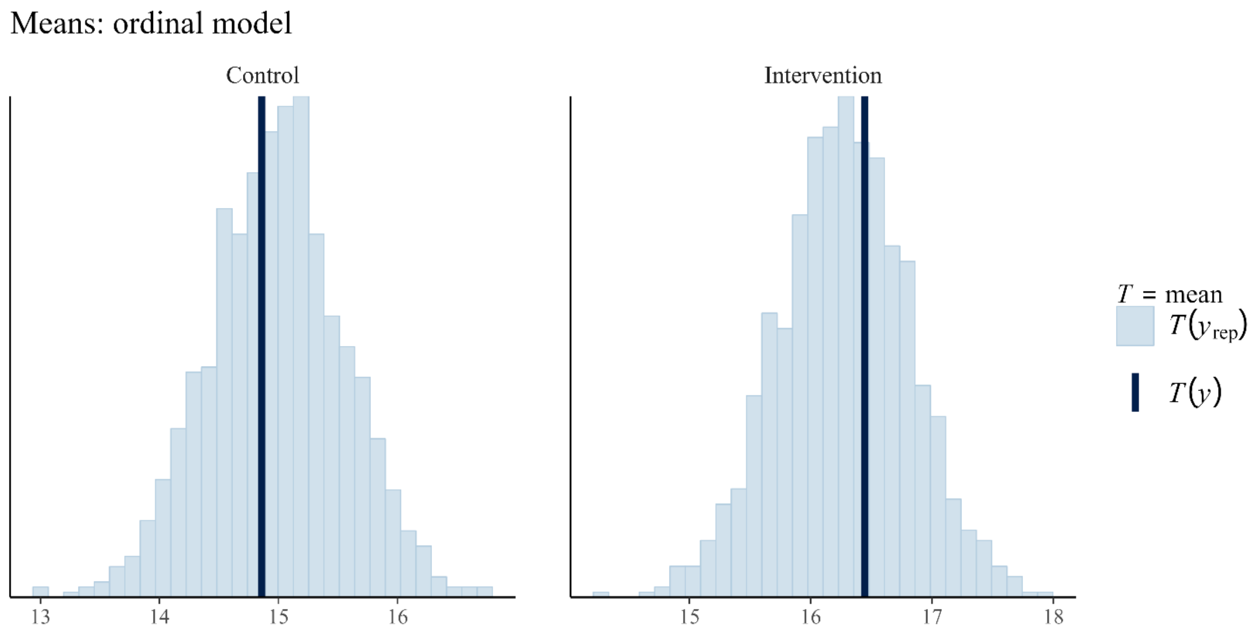

Figure S10. Posterior predictions from the ordinal (cumulative/proportional odds logistic regression) model, densities in each group, -1 predicted for worst category

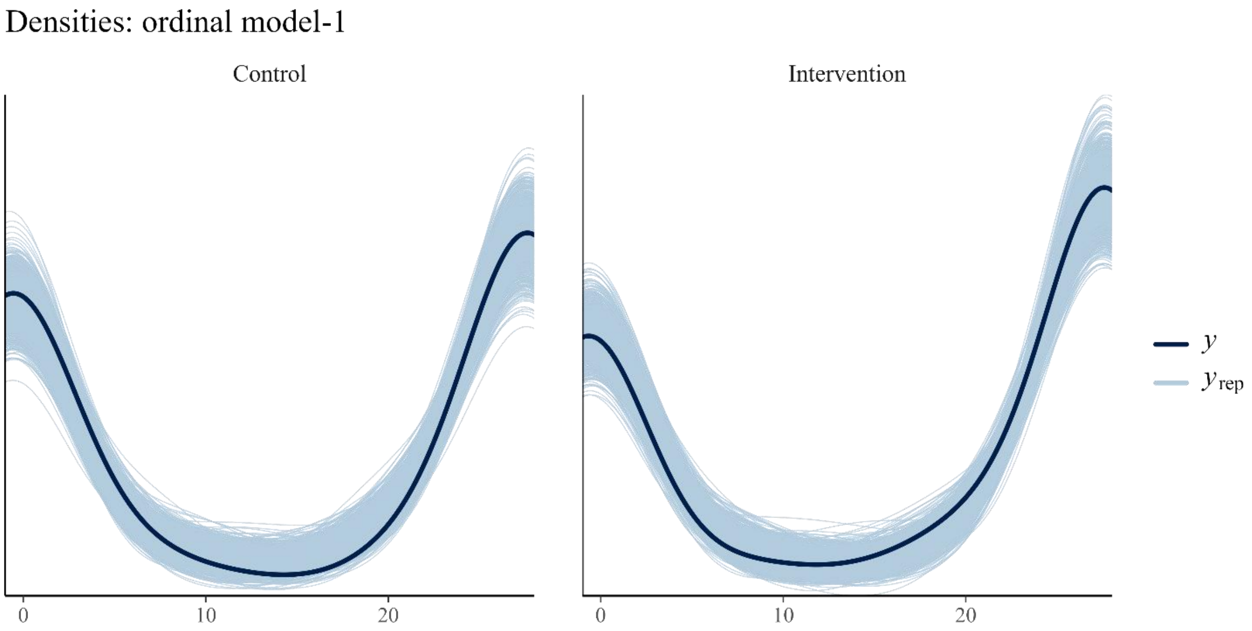

Figure S11. Posterior expected means from the ordinal (cumulative/proportional odds logistic regression) model, means in each group, -1 predicted for worst category

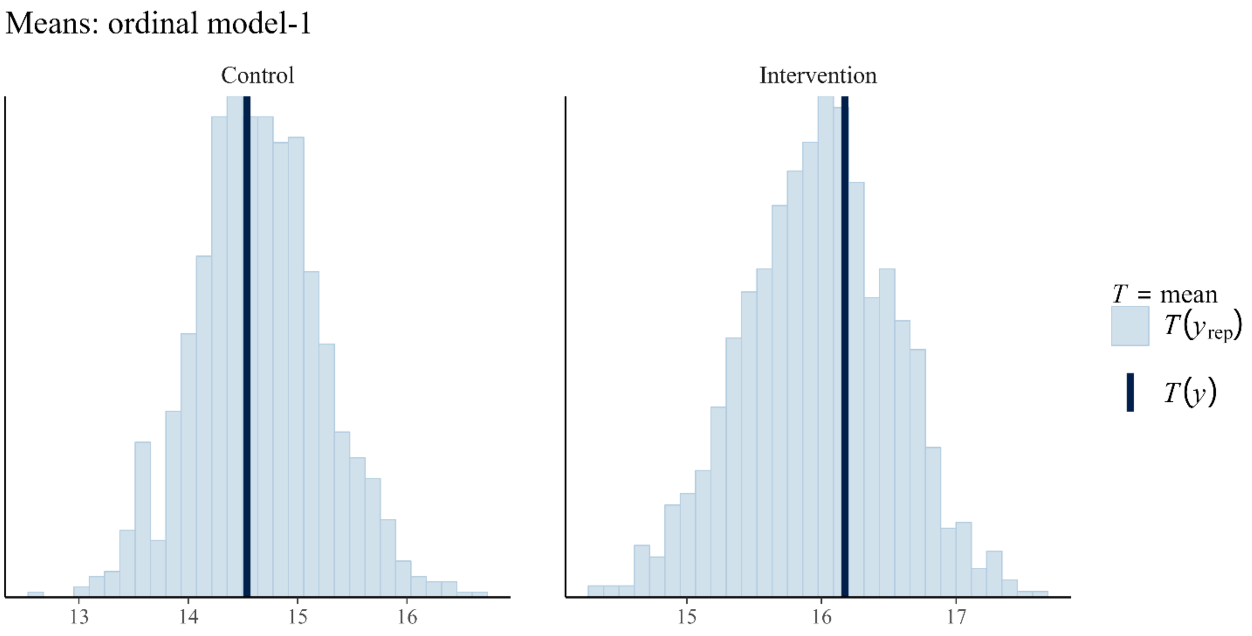

References

1. Nørskov AK, Lange T, Nielsen EE, Gluud C, Winkel P, Beyersmann J, et al. Assessment of assumptions of statistical analysis methods in randomised clinical trials: the what and how. BMJ Evid Based Med. 2021;26(3):121-126.

2. Herdman M, Gudex C, Lloyd A, Janssen M, Kind P, Parkin D, et al. Development and preliminary testing of the new five-level version of EQ-5D (EQ-5D-5L). Qual Life Res. 2011;20(10):1727-1736.

3. Writing Committee for the REMAP-CAP Investigators. Effect of Hydrocortisone on Mortality and Organ Support in Patients With Severe COVID-19: The REMAP-CAP COVID-19 Corticosteroid Domain Randomized Clinical Trial. JAMA. 2020;324(13):1317-1329.

4. Zeileis A, Kleiber C, Jackman S. Regression models for count data in R. J Stat Softw. 2008;27(8):1-25.

5. Liu F, Kong Y. zoib: An R package for Bayesian inference for Beta Regression and Zero/One Inflated Beta Regression. R J. 2015;7(2):34-51.

6. Ospina R, Ferrari SLP. A general class of zero-or-one inflated beta regression models. Comput Stat Data Anal. 2012;56(6):1609-1623.

7. Harrell FE. Regression Modeling Strategies. 2nd ed. New York: Springer; 2015.

8. Bürkner P-C, Vuorre M. Ordinal Regression Models in Psychology: A Tutorial. Adv Methods Pract Psychol Sci. 2019;2:77-101.

9. Harrell F. Violation of Proportional Odds is Not Fatal. Statistical Thinking. 2020. Available from: https://www.fharrell.com/post/po/. Accessed 07 Feb 2023.

10. Gabry J, Simpson D, Vehtari A, Betancourt M, Gelman A. Visualization in Bayesian workflow. J R Stat Soc A. 2019;182:389-402.

11. COVID STEROID 2 Trial Group. Effect of 12 mg vs 6 mg of Dexamethasone on the Number of Days Alive Without Life Support in Adults With COVID-19 and Severe Hypoxemia. JAMA. 2021;326(18):1807-1817.
